# Human Beta Oscillations Reflect Magnitude and Fidelity of Priority Shifts in Working Memory

**DOI:** 10.1523/JNEUROSCI.1548-25.2026

**Published:** 2026-02-06

**Authors:** Nicholas E. Myers, Mark G. Stokes, Paul S. Muhle-Karbe

**Affiliations:** ^1^ School of Psychology, University of Nottingham, Nottingham NG7 2RD, United Kingdom; ^2^ Centre for Neurotechnology, Neuromodulation and Neurotherapeutics, University of Nottingham, Nottingham NG7 2RD, United Kingdom; ^3^ Department of Experimental Psychology, University of Oxford, Oxford OX1 3EL, United Kingdom; ^4^ School of Psychology, University of Birmingham, Birmingham B15 2TT, United Kingdom; ^5^ Centre for Human Brain Health, University of Birmingham, Birmingham B15 2TT, United Kingdom

**Keywords:** attention, beta oscillations, EEG, multivariate pattern analysis, task switching, theta oscillations, working memory

## Abstract

Flexible prioritization in working memory (WM) is supported by neural oscillations in frontal and sensory brain areas, but the roles of different oscillations remain poorly understood. Recordings in humans suggest an interplay between prefrontal slow frequency (2–8 Hz) and posterior alpha-band (10 Hz) oscillations regulating top-down control and retrieval of WM representations, respectively. Complementary work, primarily in nonhuman primates, suggests an additional role for beta (15–30 Hz) oscillations in clearing or inhibiting stimuli from entering WM. Here we investigated the role of neural oscillations in prioritizing WM content using electroencephalography (EEG) as participants (humans of any sex) performed a task requiring frequent priority switches between two memorized oriented bars. Behavioral performance revealed switch costs, which scaled with the angular distance between the two items, suggesting that priority shifts are modulated by shift magnitude. Time–frequency analyses revealed increased frontal theta (4–8 Hz) and decreased central-parietal beta (15–25 Hz) power during switches. Crucially, only beta power scaled with the magnitude of the priority shift and predicted the fidelity of neural decoding of the newly prioritized item during subsequent recall. Theta power, in contrast, was elevated on switch trials but did not vary with update magnitude or decoding strength, suggesting a more general role in signaling control demands. Our findings highlight a particular and previously overlooked role for beta-band oscillations in the flexible prioritization of WM content.

## Significance Statement

Working memory permits flexible switching between mental representations, so we can focus on what is most relevant at the moment. Different brain rhythms in frontal control and sensory memory storage areas orchestrate switches but their respective roles remain unclear. Here, using EEG, we show that power reductions of ∼20 Hz oscillations over central-parietal regions, usually associated with the motor system, closely track the magnitude of the required switch and the fidelity of the prioritized memory. In contrast, slower 4–8 Hz (theta-band) activity over frontal regions increases during priority switches but tracks neither magnitude nor fidelity. Our findings suggest a unique function for central-parietal beta oscillations in the flexible control of working memory.

## Introduction

Working memory (WM) supports the maintenance of task-relevant information, but its capacity is severely limited. Focusing attention on individual items in WM permits effective use of this limited capacity, allowing us to remember more quickly and accurately (Griffin and Nobre, 2003; Souza and Oberauer, 2016; Van Ede and Nobre, 2023). Multiple-state models (Cowan, 2000; Oberauer, 2002) propose that high-priority items are stored in a privileged state that guides perception and action, while other items are concurrently held in a deprioritized accessory state without driving behavior (Olivers et al., 2011).

Neurally, prioritized and deprioritized WM items may be represented in different brain areas (Christophel et al., 2018) and representational formats (van Loon et al., 2018; Yu et al., 2020; Iamshchinina et al., 2021; Panichello and Buschman, 2021; El-Gaby et al., 2024; Paluch et al., 2025). Remarkably, items can be flexibly moved in and out of the focus of attention (Rerko and Oberauer, 2013; Rerko et al., 2014; Muhle-Karbe et al., 2021). Yet the neural mechanisms that underpin priority switches remain to be delineated. This is the focus of the current study.

Prefrontal neural synchronization for controlled access to items stored in sensory brain areas (Wallis et al., 2015) may represent such a mechanism. More specifically, low-frequency oscillations in the delta and theta frequency ranges (2–8 Hz) originating in frontal cortex may play a role in orchestrating top-down control over WM representations stored in sensory or medial temporal areas (Liebe et al., 2012; Roux and Uhlhaas, 2014; Johnson et al., 2018; Berger et al., 2019). Retrieval of these sensory WM representations may occur via spatiotopic desynchronization of alpha-band oscillations (Poch et al., 2014; Myers et al., 2015b; van Ede et al., 2016; de Vries et al., 2017, 2018; Riddle et al., 2020, 2024).

Parallel work has emphasized the role of beta oscillations (15–30 Hz) in WM (Lundqvist et al., 2016; Spitzer and Haegens, 2017; Miller et al., 2018; Liljefors et al., 2024) but their role in prioritization is less clear, as priority switches likely require multiple subprocesses that could be linked to beta: a shift in beta synchrony could signal a change in task context (Engel and Fries, 2010) or facilitate removal of the previously prioritized memory (Lundqvist et al., 2018). Given the role of beta in coordinating cortical-basal ganglia loops (Jenkinson and Brown, 2011; Brittain and Brown, 2014), which have been implicated in gating information in and out of WM (D’Ardenne et al., 2012; Chatham et al., 2014; Chatham and Badre, 2015), they may alternatively play a role in selecting a new memory into the prioritized state.

Prioritization in WM is typically investigated with the retro-cue paradigm, where participants initially encode multiple memory items until a subsequent cue signals which item will be task-relevant for a forthcoming decision (Griffin and Nobre, 2003). This procedure typically incurs only a single priority shift and therefore confounds changes in priority states with changes in memory load, as the initially prioritized item can be dropped after a switch. We recently developed a paradigm that avoids this issue by requiring frequent priority switches between the same two items, so that neither can be dropped from memory. Using this paradigm, we have previously shown that both prioritized and deprioritized memory items can be decoded from electroencephalography (EEG) recordings and that they track different aspects of memory-guided behavior (Muhle-Karbe et al., 2021).

Here, we sought to leverage this dataset to characterize the role of theta and beta oscillations during priority switches. We related both oscillations to the magnitude of priority switches and their fidelity by harnessing single-trial decoding of prioritized and deprioritized memory items. Priority switches increased frontal theta and decreased central-parietal alpha and beta power. We found that beta (but not theta or alpha) oscillations correlated with the magnitude of priority switches and the decoding strength of newly prioritized memory items, but not the previously prioritized item, suggesting that beta oscillations are primarily involved in prioritizing new items.

## Materials and Methods

### Participants

We reanalyzed data from a previously published study (Muhle-Karbe et al., 2021). Forty-three adults participated in total. We combined data from two closely related variants of the same visual WM task (Experiments 1 and 2). Our previous study showed identical results across both variants. We therefore collapsed all analyses across both experiments. Twenty adults participated in Experiment 1 (mean age = 28.1, age range = 18–37, 10 female, 10 male, 1 left-handed, 19 right-handed) and 30 participated in Experiment 2 (mean age = 26.8, age range = 19–41, 14 female, 16 male, 2 left-handed, 28 right-handed). Seven participants took part in both experiments, yielding a final sample of 43 participants. For participants who completed both experiments, we analyzed data separately within each experiment before averaging the results across the two experiments. All participants reported normal or corrected-to-normal vision and were compensated for their participation. The study was approved by the Central University Research Ethics Committee of the University of Oxford.

### Apparatus

Stimuli were presented using the Psychophysics Toolbox (Brainard, 1997) on a 22″ monitor with a refresh rate of 100 Hz, viewed at a distance of ∼60 cm in an electrically shielded, sound-attenuated booth with dim background lighting. Stimuli appeared on a gray background ([127 127 127]). Participants responded with left and right index fingers on the “B” and “Y” buttons of a QWERTY keyboard (placement counterbalanced across participants).

### EEG acquisition

EEG data were collected with a NeuroScan SynAmps RT Amplifier and Curry 7 software. Sixty-one EEG channels were placed following the extended 10–20 positioning system. Channel impedances were kept below 5 kOhm. In both studies, eye movements were recorded via electrooculography (EOG) using electrodes placed above and below the left eye and to the outside of each eye. We also recorded activity in the first dorsal interosseus muscle of the left and right hand via electromyography (EMG). Additional electrodes were placed on the left and right mastoids, which were averaged and used for offline rereferencing. The ground electrode was placed on the left elbow. Data were recorded at 1,000 Hz.

### Eye-tracking

In some participants (Experiment 2, 30 participants), we additionally recorded eye movements using a remote infrared eye-tracker (SR Research, EyeLink 1000) sampling both eyes at 1,000 Hz. These data were used to confirm that participants maintained close fixation and that EEG-based decoding of memory contents could not be explained by systematic deviations in fixation location (see Muhle-Karbe et al., 2021, for control analyses). Eye-tracking data were not further analyzed here.

### Task design

**Figure 1.**
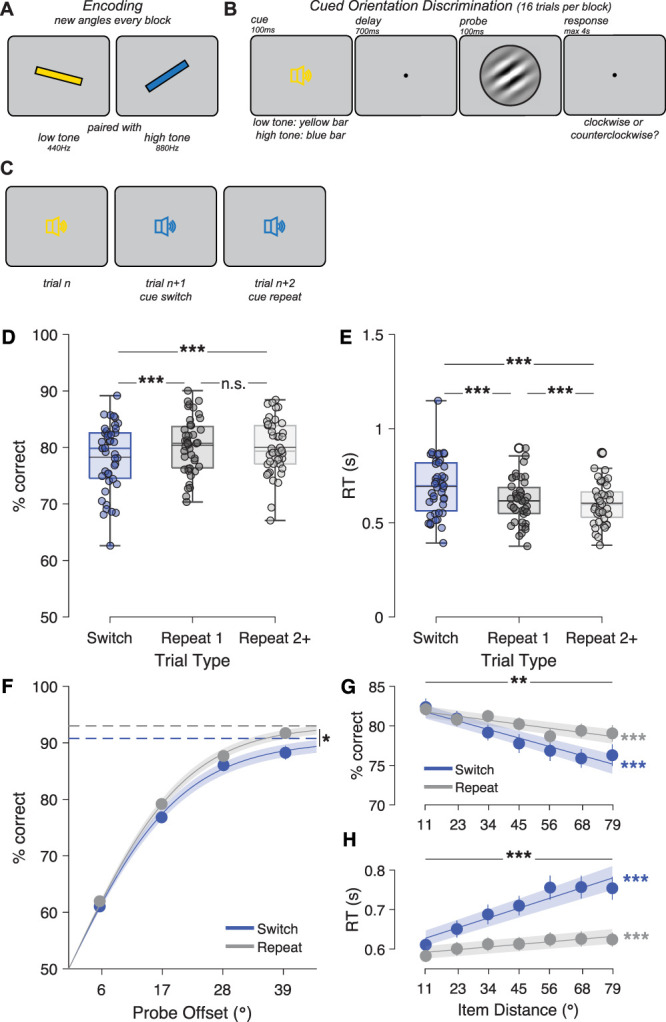
Shifting priority between WM contents incurs a temporary switch cost. ***A***, Each block began with encoding of two oriented bars. Each bar was associated with an auditory cue. ***B***, On each trial, a cue indicating which WM item was relevant was followed by a probe. Participants judged the probe as clockwise or counterclockwise relative to the cued memory orientation. ***C***, Successive trials could cue either the same or different items, requiring frequent priority switches and maintenance of both items throughout the block. ***D***, Accuracy was lower on Switch trials than the first Repeat trial after a switch or subsequent Repeat trials (max. 7 repetitions). Colored horizontal lines show median, and black horizontal lines show mean. Dots are individual observers. ***E***, Reaction time was slowest on Switch trials and slightly slower on the first Repeat trial. Empty black circles (both Repeat conditions) represent one outlier. ***F***, Psychometric curves fit to accuracy had comparable slopes but higher guess rates on Switch trials. Dots are data, with error bars indicating standard error of the mean (SEM). Line is the mean psychometric curve fit (shaded area around mean shows SEM). Horizontal dashed lines are the mean asymptotic performance (“guess rate”) for each condition (blue, Switch; gray, Repeat). ***G***, The similarity between WM orientations (“Item Distance”) affected accuracy, especially on Switch trials, and accounted for the switch cost. Lines are mean of linear fits to each observer and condition (shaded area shows SEM). ***H***, Item distance increased reaction time more on Switch than Repeat trials. ***p < 0.001, **p < 0.01, *p < 0.05, n.s., not significant.

The experiment used a 2-item visual working memory task requiring frequent priority switches (Fig. 1*A*). Task blocks consisted of 16 trials. At the beginning of each block, participants encoded in WM two orientated bars (6° visual angle in length and 0.25° in width, presented at the same location as subsequent stimuli) in blue ([25.5 25.5 204]) and yellow ([204 204 25.5]). These bars served as memory items for the remainder of the block. The two orientations were drawn randomly from a set of 16 possible orientations (spaced evenly at 11.25° intervals from 2.8125° to 171.5625°) with the constraint that the two items could not be identical or exactly orthogonal. As a result, the absolute angular distance (“item distance”) between them ranged from 11.25° to 78.75°. One bar color was associated with a high-pitch tone and the other with a low-pitch tone (mapping counterbalanced across participants), with the two tones serving as retrieval cues for the respective bars later in the block. Participants had unlimited time to encode the two bars and initiated the block via button press.

Within the block, each trial started with a presentation of an auditory cue (pure sinusoidal tones, low tone, 440 Hz; high tone, 880 Hz; duration, 100 ms including a 10 ms ramp-up and 10 ms ramp-down). The tone signaled which memory item should be used for a forthcoming perceptual decision (cued item), while the other item was maintained for later use in the block (uncued item). The cue was followed by a 700 ms delay period within which a black fixation dot was presented centrally on the screen (0.15° diameter). A randomly oriented Gabor patch was presented (orientation drawn from 16 possible orientations, spaced evenly at 11.25° intervals from 8.4375 to 177.1875°; patches had 6° diameter, 50% contrast, 1.75 cycles/°, random phase, and a Gaussian envelope with 1.2° SD). Participants were given a maximum of 4,000 ms to judge the probe via button press as clockwise or counterclockwise relative to the cued memory orientation. Probes were presented for 100 ms and replaced by a fixation dot for the remainder of the response period. In Experiment 1, the probe was presented centrally on the screen on all trials. In Experiment 2, the probe was presented laterally at a distance of 6° from the screen center. The side of probe presentation (left vs right) alternated predictably across blocks. A noise patch (Gaussian smoothed random white noise using a kernel with 0.13° SD, convolved with a Gaussian envelope with 1.2° SD) that matched the probe stimulus in luminance, size, contrast, and eccentricity was presented on the side of the screen at which no probe appeared. Clockwise and counterclockwise decisions were indicated with the right and left index fingers, respectively. The response period was followed by a variable intertrial interval (400–900 ms, drawn from a truncated exponential distribution with mean 550 ms).

The next trial cued either the other item (Switch trial) or the same item again (Repeat trial). The likelihood of a cue repeat on each trial was drawn from a modified exponential distribution with a minimum of 1 repeat and a maximum of 7 repeats after each Switch trial. This resulted in comparable trial numbers for Switch trials (464 ± 9, mean ± SEM), the first Repeat trial after a switch (Repeat 1, 553 ± 9), and subsequent Repeat trials (Repeats 2–7, abbreviated Repeat 2+, 646 ± 10). Participants received feedback only at the end of each block when their mean accuracy and response time were presented. Accordingly, they had to maintain precise representations of both memory items for the whole duration of the block, as they could not rely on trial-wise feedback to infer the orientation of an item if it was forgotten. Overall, participants completed 128 blocks, resulting in a total of 2,048 trials and lasting ∼2 h.

### Behavioral analysis

We were primarily interested in the effect of priority switches, i.e., trials in which the item cued as relevant by the auditory cue was not the same as the item cued on the preceding trial (Switch trials). We compared Switch trials with Repeat trials, i.e., trials in which the same item was cued as on the preceding trial. We excluded the first trial in each block because it was neither a switch nor a repeat trial. We then calculated mean accuracy and reaction time on switch trials, the first repeat trial (Repeat 1), and any subsequent repeat trials (Repeat 2+). Reaction times were calculated as the median of all correct responses to a given condition. Since accuracy on Repeat 1 and Repeat 2+ trials was largely the same (Fig. 1*A*), for the remainder of the manuscript, we focused on comparing Switch with Repeat 1 trials. Including Repeat 2+ trials in any analyses did not materially affect our results.

We next fit a psychometric curve to the behavioral data to separately estimate the memory precision (inverse of the slope, *σ*) and the lapse rate (*λ*) using the response function:

P(response=clockwise|θ)=(1−λ)Φ(θ,μ,σ)+λ,

where *θ* is the angular difference between the probe and cued memory stimulus, *λ* is the lapse rate, *μ* is the response bias (fixed to 0), *σ* is the inverse of the memory precision, and Φ is the cumulative density function of the normal distribution.

Precision (1/*σ*) and lapse rate (*λ*) were fit using maximum likelihood estimation separately for Switch and Repeat trials. Estimated parameters were compared between conditions using pairwise *t* tests.

We extended the psychometric model to include erroneous comparisons to the uncued item (“swap errors”):

$$P(\mathrm{response}=\mathrm{clockwise}\;|\theta_{\mathrm{cued}},\theta_{\mathrm{uncued}})=(1-p_{\mathrm{swap}}-\lambda )\Phi (\theta_{\mathrm{cued}},\mu ,\sigma )+p_{\mathrm{swap}}\Phi (\theta_{\mathrm{uncued}},\mu ,\sigma )+\lambda ,$$

where *θ*_cued_ is the angular difference of the probe orientation relative to the cued WM item, *θ*_uncued_ is the angular difference of the probe orientation relative to the uncued WM item, and *p*_swap_ is the swap rate.

To examine the effect of item distance, we fit a linear model of item distance (with values ranging from 11.25 to 78.75°) to accuracy and reaction time data on Switch and Repeat trials separately. Regression coefficients were then compared against zero and each other using one-sample or paired-samples *t* tests.

### EEG preprocessing

EEG data were rereferenced to the average of both mastoids. EEG, EOG, and EMG data were downsampled to 250 Hz and bandpass-filtered between 0.1 and 45 Hz. EEG channels with excessive noise were identified through visual inspection and replaced via interpolation using a weighted average of the surrounding electrodes. The continuous data were epoched from 750 ms before to 2,500 ms after the onset of the cue on each trial. Each trial was inspected visually for blinks, eye movements, and nonstereotyped artifacts. Trials were rejected if they contained any of those artifacts during the cue-probe delay or probe period. Stereotyped artifacts (from blinks, saccades, or muscle artifacts) outside those periods were subsequently removed via independent component analysis. Unless stated otherwise, the data were baseline-corrected for the decoding analyses using the average signal from the time window of 200–50 ms before cue onset.

### EEG decoding

We conducted multivariate pattern analysis to characterize the neural representations of the cued and uncued WM item. We followed a previously established approach (Wolff et al., 2020a,b; Muhle-Karbe et al., 2021) that used the signal at multiple posterior EEG sensors, pooled across multiple timepoints, to decode remembered orientations. This approach exploits the dynamic temporal structure of event-related potentials by pooling multivariate information in time, capturing information encoded in spatial activation patterns and in the temporal unfolding of these patterns to achieve greater decoding sensitivity (Wolff et al., 2020a).

In keeping with this previous work, decoding analyses were conducted only within posterior EEG sensors (P7, P5, P3, P1, P2, P4, P6, P8, PO7, PO5, PO3, POz, PO4, PO8, O1, Oz, O2). We selected these channels to be consistent with previous studies and because orientation signals are typically expressed most strongly in posterior regions (Cichy et al., 2015; Myers et al., 2015a; Wolff et al., 2020a). We pooled the signal across a 450 ms backward-looking sliding window (i.e., decoding at 800 ms after cue onset used data from 350 to 800 ms), downsampled to 83.3 Hz (i.e., one timepoint every 12 ms), yielding 37 timepoints from each of the 17 EEG sensors, or 629 features in total. Data were demeaned across timepoints within each sensor (to reduce any potential carryover of decodable signal from other time windows, although this step seemed to have a minimal effect in our data, see Fig. S5 top vs bottom row). Our previous work showed that WM decoding peaked around the time of the decision, ∼200–400 ms after probe onset. We therefore focused on the postprobe window for analysis, repeating the decoding analysis in a sliding window from 800 to 1,400 ms relative to cue onset (or 0–600 ms relative to probe onset), in 50 ms steps.

We computed Mahalanobis distances between the patterns of sensor activity that were evoked by different stimulus orientations and measured the extent to which these distances reflected the underlying circular orientation space. We used a 13-fold cross-validation procedure. On each fold, data from 10 task blocks served as test data and all the remaining data served as training data. From the training data we calculated the average response to each of the 16 orientations, yielding a *N*_orientations_-by-*N*_features_ (16 × 629) training data matrix *M*_train_. Mahalanobis distances between the 16 average patterns in the training data and each test trial *i* (*M_i_*, 1 × 629) were then computed, using the noise covariance from the training data. Noise covariance was calculated on the residual data after subtracting orientation-specific mean activity from each trial, using a shrinkage estimator (Ledoit and Wolf, 2004). Smaller values indicated a smaller Mahalanobis distance, i.e., greater similarity between the test trial and a given orientation from the training set. For each test trial, this yielded a vector *D_i_* of 16 distances. *D_i_* was circularly shifted to center the vector on the test trial orientation *θ_i_* and inverted so larger values indicated greater pattern similarity, yielding a “tuning curve” that peaks at the center of the curve when the orientation is decodable. To calculate a trial-wise index of decoding, we computed a summary measure that estimated how closely the tuning curve resembled a cosine peaking at the test trial orientation. This index was calculated as the cosine vector mean of the 16-dimensional tuning curve by multiplying each distance with the cosine of the orientation difference to the test trial (Sprague et al., 2016; Wolff et al., 2017):

$$\mathrm{decodin}g_{i}\;=\;\overset{16}{\underset{\;j=1}{\sum }}\;\cos\;(2\theta_{j})D_{i,j},$$

where *θ_j_* is the orientation offset of the *j*th point in the tuning curve (ranging from −78.75 to +90°) and *D_i_*_,*j*_ is the Mahalanobis distance between test trial *i* and the training set average for the orientation at *θ_j_* relative to *θ_i_*. Angles were doubled in the equation to account for the 180° symmetry of the orientation stimuli. Because the cosine is zero centered, if the tuning curve is flat, this operation yields a mean decoding score of 0. If decoding peaks at the center of the curve (at *θ_j_* = 0°), this yields a positive decoding score. An inverted curve (peak near 90°) would yield a negative decoding score. The absolute value of the decoding score is difficult to interpret because it depends on the voltage change over time, the number of sensors, and the number of timepoints. The analysis was run separately for the cued and the uncued orientation. In Experiment 2, decoding analyses were conducted separately for blocks with probe presentation on the left and right side, with training and test data matched by side. Training data consisted of all available trials, with decoding accuracy of the test data split afterwards into Switch and Repeat trials.

### EEG time–frequency analysis

Preprocessed EEG data were transformed with a spatial Laplacian filter to reduce spatial spread of neural sources across the scalp. We used the FieldTrip function *ft_scalpcurrentdensity* [using default conductivity (0.33) and lambda (10^−5^) values and a degree of 10 as in de Vries et al. (2018)]. We then calculated the time–frequency transform in sliding windows from −0.75 s before cue onset to 2.5 s after cue onset (in 40 ms steps). At each timepoint, we calculated power at frequencies between 2 and 40 Hz (in 1 Hz steps) using Hanning tapers (using a time window corresponding to five cycles of the frequency of interest centered on the current timepoint). We then log-transformed power (10 × log10) and baseline-corrected using power from 0.75 to 0.25 s before cue onset.

### Statistical testing

Unless otherwise noted, we used multiple linear regression to establish relationships between oscillatory power and other task variables (item distance, WM decoding), separately for Switch and Repeat trials. Regressors and dependent variables were *z*-scored prior to regression. We tested for significance using one-sample *t* tests against 0 (at each timepoint, frequency, or time–frequency combination) and corrected for multiple comparisons via cluster-based permutation testing using 10,000 permutations (Maris and Oostenveld, 2007).

### Data and code availability

Data is available at https://gin.g-node.org/nicholas.myers/Beta-Shifts-Working-Memory/. Analysis code is available at https://github.com/nemyers/Beta-Shifts-Working-Memory.

## Results

### WM priority switches incur a temporary behavioral cost

Forty-three participants performed a visual WM task designed to induce frequent priority switches while we recorded their brain activity through electroencephalographic (EEG) recordings (see Materials and Methods for details). The task consisted of 128 miniblocks each of which contained 16 trials. Participants first encoded two randomly oriented bars into WM and maintained both for the duration of the mini-block. Each bar was associated with a different auditory cue (Fig. 1*A*). Every trial began with an auditory cue indicating which of the two items was relevant (Fig. 1*B*). Participants therefore prioritized the cued stimulus and deprioritized the other (uncued) stimulus. After a 700 ms cue-probe interval, a randomly oriented probe stimulus appeared and was judged as clockwise or counterclockwise relative to the cued WM orientation. The following trial could then either repeat the same cue (Fig. 1*C*, Repeat trials, max. 7 repeats in a row) or switch to the other auditory cue (Switch trials), requiring prioritization of the previously uncued item. Multiple switches occurred in each block, so participants had to keep both stimuli in WM.

Accuracy on Switch trials (78.3% ± 0.9% SEM; Fig. 1*D*) was modestly but significantly lower than on the first Repeat trial (80.4 ± 0.7%, *t*_(42) _= −4.41, *p* = 7.01 × 10^−5^, Cohen's *d* = −0.67) and lower than on subsequent repeat trials (80.0 ± 0.7%, *t*_(42) _= −4.06, *p* = 2.08 × 10^−4^, *d* = −0.62). The first Repeat trial did not differ from subsequent repeats (*t*_(42)_ = 1.11, *p* = 0.275, *d* = 0.17). Switch costs were driven by an increase in guess rate on Switch trials (9.2 ± 1.1%; Fig. 1*F*) compared with all Repeat trials (7.0 ± 0.7%, *t*_(42)_ = 2.41, *p* = 0.021, *d* = 0.37). In contrast, precision remained unchanged (Switch: 18.2 ± 1.2°, Repeat: 17.6 ± 0.7°, *t*_(42)_ = 0.43, *p* = 0.67, *d* = 0.07).

Similarly, median reaction time on correct trials was significantly slower on Switch trials (693 ± 23 ms; Fig. 1*E*) compared with either the first Repeat trial (616 ± 18 ms, *t*_(42)_ = 9.19, *p* = 1.30 × 10^−11^, *d* = 1.40) or subsequent Repeat trials (604 ± 16 ms, *t*_(42)_ = 9.91, *p* = 1.47 × 10^−12^, *d* = 1.51). Reaction time on the first Repeat trial was also significantly slower than on subsequent Repeats (*t*_(42)_ = 3.71, *p* = 6.08 × 10^−4^, *d* = 0.57).

### Priority switch costs scale with update magnitude

We next explored whether the similarity between the two items held in WM affected behavior (Fig. 1*G*,*H*). As the angular distance between the cued and uncued item (“item distance,” ranging from 11 to 79° in 11.25° steps) increased, accuracy decreased on both Switch trials (decrease in accuracy of 0.97 ± 0.17% per 10°, *t* test on slope: *t*_(42) _= −5.71, *p* = 1.04 × 10^−6^, *d* = −0.87) and Repeat trials (0.46 ± 0.11% per 10°, *t*_(42) _= −4.16, *p* = 1.54 × 10^−4^, *d* = −0.63). However, the effect of item distance was stronger on Switch trials *t*_(42) _= −3.00, *p* = 0.0045, *d* = −0.46). After regressing out item distance, there was no longer a significant switch cost (*t*_(42)_ = 0.73, *p* = 0.47, *d* = 0.11), indicating that switch costs were driven primarily by trials requiring a large update from the previous to the currently cued item. Consistent with this, blocks with similar WM item orientations (item distance <25°) showed no switch cost on accuracy (*t*_(42)_ ≤ 0.29, *p* ≥ 0.77, *d* ≤ 0.04).

We found complementary effects on reaction time (Fig. 1*E*), which increased significantly with item distance on Switch trials (22.5 ± 2.6 ms per 10°, *t*_(42)_ = 8.64, *p* = 7.24 × 10^−11^, *d* = 1.32) and, to a lesser degree, on Repeat trials (5.9 ± 0.9 ms per 10°, *t*_(42)_ = 6.60, *p* = 5.4 × 10^−8^, *d* = 1.01). Again, the effect of item distance was significantly larger on Switch trials (*t*_(42)_ = 7.69, *p* = 1.5 × 10^−9^, *d* = 1.17). However, even after regressing out the effect, a significant difference between Switch and Repeat trials remained (*t*_(42)_ = 2.31, *p* = 0.026, *d* = 0.35). Therefore, reaction time switch costs were present even in blocks with very similar WM items (item distance 11°: *t*_(42) _= −3.89, *p* = 3.53 × 10^−4^, *d* = 0.59).

The item distance effect could have been driven in part by occasional failures to update the cued item on Switch trials, leading to responses relative to the uncued item (sometimes referred to as a swap error; Bays et al., 2009). When we estimated the swap rate separately from the lapse rate, we indeed found that both increased on Switch trials (Suppl. Fig. S1*d*,*e*). Nevertheless, when we accounted for potential swap error effects, there was still an independent significant effect of item distance on both accuracy and reaction time (Suppl. Fig. S1*f*,*g*).

### Priority switches lead to temporary decreases in beta-band power and sustained increases in theta-band power

We next examined the neural oscillatory correlates of priority switches in WM. We focused on oscillations because previous work has implicated frontal slow oscillations in the delta/theta-band and posterior alpha-band power reductions (Poch et al., 2014; Wallis et al., 2015; de Vries et al., 2018; Riddle et al., 2020) in switching attention between items held in WM. Here, we observed increased frontal theta power and decreased posterior alpha power but additionally found a pronounced reduction of power in the beta-band (15–25 Hz).

**Figure 2.**
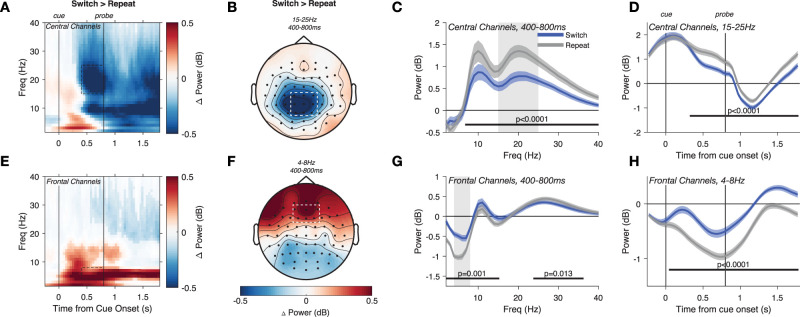
Priority switches led to a temporary decrease in beta-band (15–25 Hz) and an increase in theta-band (4–8 Hz) power. ***A***, Average difference in power between Switch and Repeat trials at central channels. Between the cue and probe onset, power reduction on Switch trials was strongest in the 15–25 Hz range but extended from ∼7 to 40 Hz (shading indicates significant cluster from permutation test). Time–frequency points are plotted with saturated colors if they belong to a significant cluster (permutation test, *p* < 0.05). ***B***, Beta power reduction on Switch trials was strongest at central-parietal channels. ***C***, In the late cue period (400–800 ms after cue onset), power at central channels peaked in the alpha and beta bands and was lower on Switch trials from 7 to 40 Hz. Shaded area around mean indicates SEM, and black bar denotes significant difference between Switch and Repeat trials after cluster-based permutation testing. ***D***, Central 15–25 Hz power was lower throughout Switch trials but strongest in the late cue period. ***E–H***, 4–8 Hz power at frontal channels was stronger on Switch trials.

Beta-band power was significantly lower on Switch trials compared with Repeat trials (Fig. 2*A*). The effect was strongest in a frequency-limited band (∼15–25 Hz) and peaked in a brief period shortly after the onset of the cue (roughly 400–800 ms after cue onset) but before the onset of the memory probe (at 800 ms after cue onset). The effect (i.e., decreased power) was most pronounced at central-parietal sites (Fig. 2*B*) and all subsequent analyses focused on these channels (“Central Channels”: C1, Cz, C2, CP1, CPz, CP2, P1, Pz, P2). The power decrease on Switch trials at central channels extended to the alpha-band (8–14 Hz) and to higher frequencies (25–40 Hz, sig. cluster 7–40 Hz, *p* < 10^−4^ after cluster-based permutation test) but showed a pronounced peak in the beta-band (Fig. 2*C*), indicating that beta oscillations are a distinct phenomenon. The effect was significant throughout the trial (from 320 ms after cue onset) but strongest in the latter half of the cue period (Fig. 2*D*).

In parallel, we observed a significant increase in theta (4–8 Hz) power on Switch trials (Fig. 2*E*) that peaked at frontal midline channels (Fig. 2*F*, “Frontal Channels”: AF3, AFz, AF4, F1, Fz, F2). The effect was limited to the theta and alpha bands (2–15 Hz, corrected *p* = 0.001; Fig. 2*G*) but accompanied by a reduction in higher-frequency power (24–36 Hz, *p* = 0.013; Fig. 2*G*). The increase in theta-band power persisted throughout the Switch trial (Fig. 2*H*). Finally, power in the alpha-band (8–14 Hz) decreased at posterior channels (O1, Oz, O2, PO7, PO3, POz, PO4, PO8; Supplementary Fig. S2*a*). We observed the same effects when comparing Switch trials to later Repeat trials (Repeat2+; Fig. S3).

### Beta (but not theta) power scales with magnitude of priority switches

**Figure 3.**
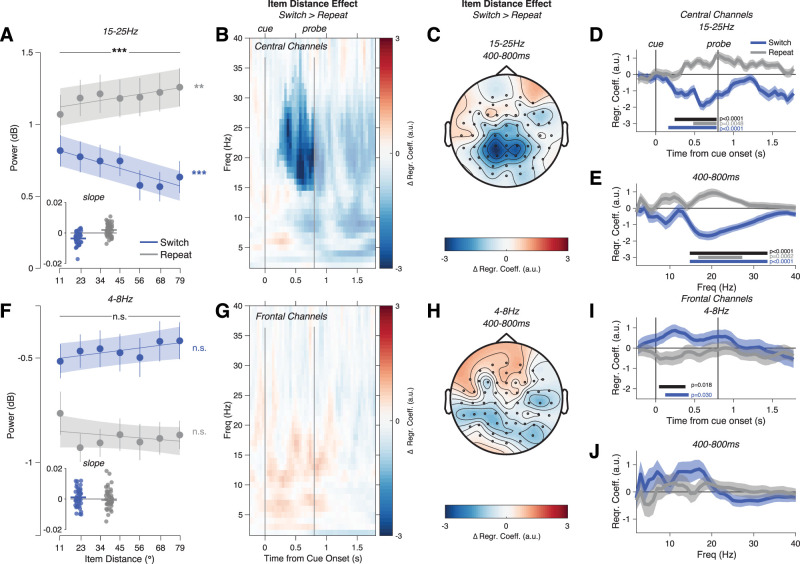
Central beta power scales with item distance. ***A***, Beta (15–25 Hz) power at central channels (400–800 ms post-cue), separated by Switch (blue) versus Repeat (gray) trials and by angular distance between WM items. A large item distance required a larger-magnitude update of the prioritized WM representation on Switch trials. Dots show average power (error bars show SEM), and line shows linear fit (shaded area is SEM). Inset shows regression coefficients for each participant. ***B***, The effect of item distance on power (regression coefficient on Switches vs coefficient on Repeats) was resolved over time and frequency and was significant primarily between 15 and 30 Hz (area with saturated color indicates significant cluster from permutation test, *p* < 0.05 corrected). ***C***, The effect peaked at central-parietal channels (compare Fig. 2*B*). ***D***, Regression coefficient resolved by time (average of 15–25 Hz power at central channels), separately for Switch and Repeat trials. Bars indicate significant effects after correction (blue, Switch vs 0; gray, Repeat vs 0; black, Switch vs Repeat). ***E***, Effect resolved by frequency (Central channels, 400–800 ms after cue onset). ***F–J***, Frontal 4–8 Hz power showed no comparable effects, except for a brief correlation on Switch trials immediately after cue onset (panel ***I***).

Given the pronounced effect of item distance on behavior, particularly on Switch trials, we tested if beta- and theta-band effects were also sensitive to item distance. Indeed, beta-band power scaled with item distance (Fig. 3*A*; *F*_(6,252)_ = 2.303, *p* = 0.035, partial *η*^2^ = 0.009), with opposing effects on Switch and Repeat trials (interaction Switch effect with Item Distance effect; *F*_(6,252)_ = 8.712, *p* = 1.25 × 10^−8^, partial *η*^2^ = 0.033). On Switch trials, larger priority switches (i.e., larger item distances) led to lower beta-band power (*t* test on linear fit, *t*_(42) _= −5.74, *p* = 9.54 × 10^−7^, *d* = −0.88). On Repeat trials, the effect was reversed, with a (smaller) increase in beta power for larger item distances (*t*_(42)_ = 3.12, *p* = 0.0033, *d* = 0.48), leading to a significant difference in slope between Switch and Repeat trials (*t*_(42) _= −5.86, *p* = 6.56 × 10^−7^, *d* = −0.89). Even after regressing out the effect of item distance, beta power was still lower on Switch trials (*t*_(42) _= −3.42, *p* = 0.0014, *d* = −0.52). The effect of item distance was confined to the beta-band (Fig. 3*B*; with band-limited sig clusters on Switch trials 15–33 Hz, *p* < 10^−4^, on Repeat trials 17–27 Hz, *p* = 0.0062, Switch vs Repeat 15–33 Hz, *p* < 10^−4^; Fig. 3*E*) and to central-parietal channels (Fig. 3*C*), emerging ∼200 ms after cue onset (Fig. 3*D*; sig. effect on Switch trials 162–782 ms, corrected *p* < 10^−4^, effect on Repeat trials 482–782 ms, *p* = 0.0048, difference Switch vs Repeat 242–782 ms, *p* < 10^−4^). Therefore, the beta-band power decrease observed on Switch trials scales with the magnitude of the update between the previous and currently cued item.

In contrast, frontal theta power was largely insensitive to item distance (Fig. 3*F*; main effect of item distance *F*_(6,252)_ = 0.343, *p* = 0.913, partial *η*^2^ = 0.001, interaction Switch by item distance *F*_(6,252)_ = 0.867, *p* = 0.519, partial *η*^2^ = 0.0034). Linear fits showed no effect of item distance in either Switch (*t*_(42)_ = 1.21, *p* = 0.233, *d* = 0.18) or Repeat trials (*t*_(42) _= −0.57, *p* = 0.574, *d* = −0.09). Theta power remained higher on Switch trials after regressing out item distance (*t*_(42)_ = 3.24, *p* = 0.0024, *d* = 0.49). We found no significant effect at frontal channels when analyzed separately by timepoint and frequency (Fig. 3*G*) or averaged over the late cue period (Fig. 3*J*). We also saw no clear theta effects at other channels (Fig. 3*H*). When we examined the effect separately for each timepoint in the trial, we did find a small effect of item distance on Switch trials immediately following the cue onset (Fig. 3*I*; effect on Switch trials from 122 to 422 ms, *p* = 0.03, difference between Switch vs Repeat effects 42–382 ms, *p* = 0.018). The early and brief effect suggests that this may reflect a phasic event-related response to the cue rather than the sustained theta oscillation increase observed on Switch trials more generally (Fig. 2*E*). We found no effect of posterior alpha power on item distance on Switch trials, with only a small effect on Repeat trials (Fig. S2*b*) that could have been driven by the spatially and spectrally adjacent beta-band effect. In sum, unlike the graded beta-band effect increasing with item distance, oscillatory theta power increases on Switch trials appear to be largely an all-or-none phenomenon.

### Beta power after priority switches predicts decoding of cued WM item

**Figure 4.**
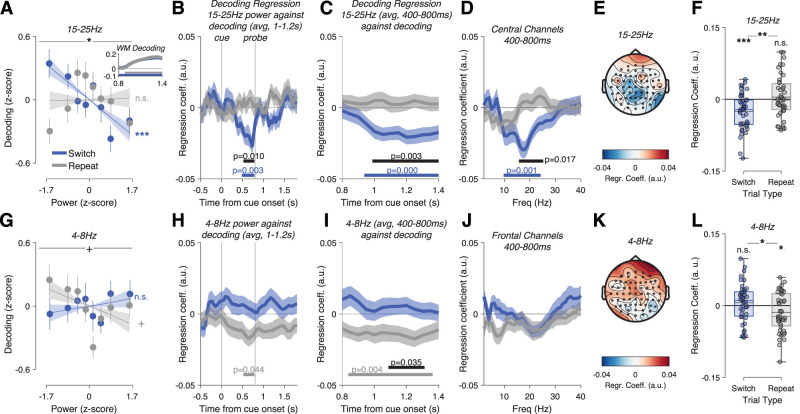
15–25 Hz power on priority switch trials predicts decoding of the newly prioritized item. ***A***, Switch trials binned by 15–25 Hz power (400–800 ms, central channels) show a linear relationship with cued orientation decoding. This is not the case on repeat trials. Dots and error bars show mean ± SEM of the data. Line and shaded area shows mean ± SEM of linear fit. Inset: Timecourse of cued orientation decoding strength (a.u.) from probe onset (0.8 s) to 600 ms after cue onset (1.4), separately for Switch and Repeat trials. ***B***, We regressed beta power across the timecourse of the trial against postprobe decoding (1–1.2 s). Only beta power in the late cue interval predicted later decoding (Switch trials only), with no effect on Repeat trials. ***C***, Switch trial beta power in the late cue period (400–800 ms) predicted decoding throughout the postprobe period, from 150 ms after probe onset (0.95 s) to the end of the probe epoch (1.4 s, 600 ms after probe onset). ***D***, Cue interval power predicted decoding in a frequency range from 10 to 24 Hz. ***E***, Topography of channel-wise power predicting decoding, peaking at central-parietal channels. ***F***, Regression coefficient of beta power on decoding from a multiple regression model including item distance and power × item distance interaction. Colored horizontal lines show median, and black horizontal lines show mean. Dots are individual observers. ***G–L***, Frontal theta power does not show this effect, but higher theta power predicts lower decoding on repeat trials (but see analysis without baseline correction; Fig. S4).

Since the magnitude of beta power reduction after priority switches scaled with the magnitude of the update, we reasoned that it might be involved in retrieving or shifting attention to the newly cued WM item. We therefore expected that beta power should predict the strength of WM decoding. To this end, we calculated a trial-by-trial orientation decoding score around the time of decision-making after the onset of the probe (200–400 ms following probe onset, i.e., 1,000–1,200 ms after cue onset). Decoding in this window was maximal (Fig. 4*A*, inset; see also Muhle-Karbe et al., 2021) and comparable on Switch and Repeat trials (Switch trials, significant decoding 800–1,400 ms after cue onset, e.g., 0–600 ms after probe onset, corrected *p* < 10^−4^, Repeat trials: significant decoding 900–1,400 ms, *p* < 10^−4^, no sig. difference between Switch and Repeat, smallest corrected *p* = 0.088).

We then related this to trial-by-trial beta-band power. When we binned all Switch trials by beta power into 8 nonoverlapping bins, we saw a clear relationship to decoding: the lower the beta-band power, the higher the decoding (Fig. 4*A*; linear regression across 8 bins, *t*_(42) _= −3.93, *p* = 0.0003, *d* = −0.60). This was not the case on Repeat trials (*t*_(42)_ = 0.14, *p* = 0.89, *d* = 0.02), leading to a significant difference between slopes (*t*_(42) _= −2.68, *p* = 0.011, *d* = −0.41). Only beta power after the switch cue and shortly before probe onset predicted decoding (Fig. 4*B*; sig. cluster 492–772 ms, corrected *p* = 0.0029, difference to Repeat trials 532–772 ms, *p* = 0.0096). Beta power in the preprobe interval (400–800 ms) predicted decoding throughout the postprobe interval (Fig. 4*C*; 950–1,400 ms after cue onset, *p* = 0.0001, difference to repeat trials 1,000–1,400 ms, *p* = 0.0033). Decoding before probe onset was much weaker and not correlated with beta power (Fig. S5). At central channels, only alpha- and beta-band power predicted decoding (Fig. 4*D*,*E*; 10–24 Hz, *p* = 0.001, sig. difference to repeat trials 16–25 Hz, *p* = 0.0172).

Because of the relationship between item distance and both beta power and accuracy, these effects could have been driven by differences in item distance. To account for this possibility, we conducted a multiple regression analysis with (*z*-scored) beta power, *z*-scored item distance and their interaction as predictors and WM decoding as the dependent variable. The effect of beta power on decoding remained significant for Switch trials (Fig. 4*F*; *t*_(42) _= −4.420, *p* = 6.82 × 10^−5^, *d* = −0.67) and nonsignificant for Repeat trials (*t*_(42)_ = 0.672, *p* = 0.506, Switch vs Repeat: *t*_(42)_ = 3.428, *p* = 0.0014, *d* = −0.52), and we saw no difference in the effect of item distance on decoding between Switch and Repeat trials (*t*_(42)_ = 1.03, *p* = 0.31 for model including 15–25 Hz power, *t*_(42)_ = 0.79, *p* = 0.43 for model including 4–8 Hz power), and there was no significant interaction between beta power and item distance on decoding (*p* > 0.22).

We saw no comparable effect of theta power on Switch trials. In fact, there appeared to be only a modest effect of theta power on decoding on Repeat trials. This effect trended into an unexpected direction, with higher theta power predicting lower decoding (Fig. 4*G*,*H*; sig. cluster 532–772 ms, *p* = 0.044, also after correcting for item distance; Fig. 4*L*). This effect occurred in a similar time range (Fig. 4*I*) as the beta power effect but appeared to be statistically weaker and showed no clear peak in the frequency spectrum (Fig. 4*J*) or strong effects at nonfrontal channels (Fig. 4*K*). More importantly, it appeared to depend on the choice of baseline and may have been driven by fluctuations in pretrial theta power. When we conducted the analysis without baseline correction (Fig. S4*b*) or after regressing baseline power out of the signal (Fig. S4*d*), there was no longer a significant relationship between theta power and decoding. Instead, increased theta power on switch trials predicted increased decoding of the cued item late in the trial, mirroring the beta power effect (Fig. S4*b*,*d*, third panel). Similarly, alpha power had no effect on decoding of the cued item with or without baseline correction (Fig. S2*c*,*f*). The beta power effect (Fig. S4*a*,*c*), on the other hand, remained unchanged and significant.

**Figure 5.**
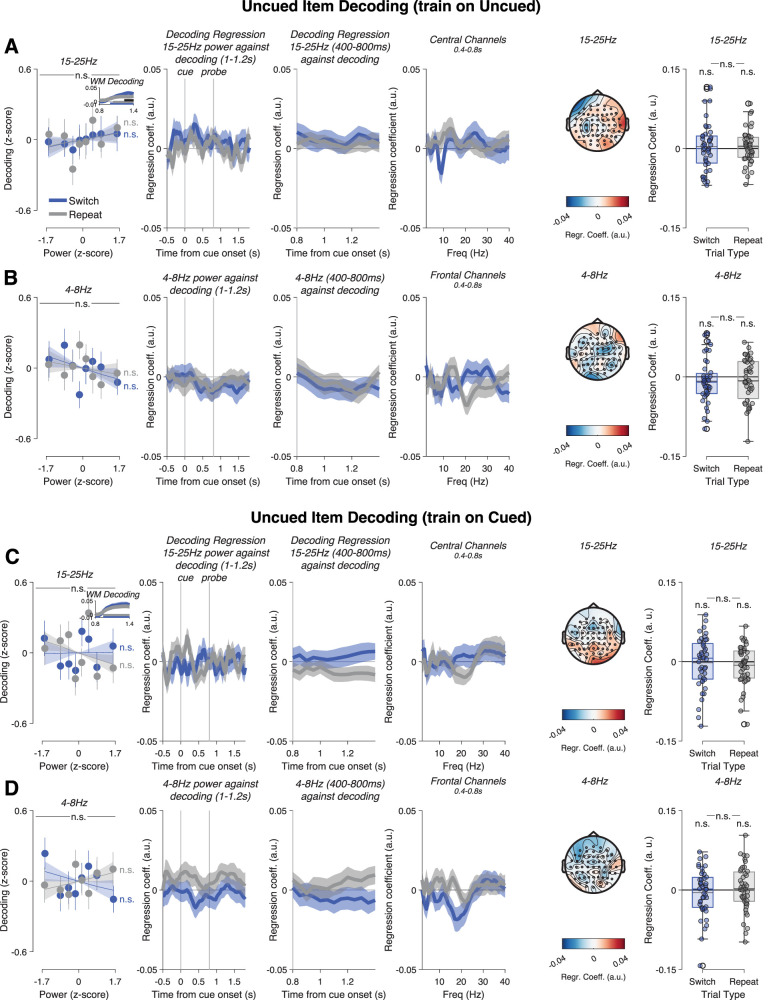
15–25 Hz and 4–8 Hz power on priority switch trials do not predict decoding of the uncued item. ***A***, No relationship between 15 and 25 Hz power and decoding of the uncued item. ***B***, Similarly, 4–8 Hz power had no effect. ***C***, ***D***, Cross-decoding the uncued item (train on cued, test on uncued) also showed no effect. Conventions as in Figure 4.

In sum, beta power had a significant effect on decoding only on switch trials, suggesting a role in prioritizing the newly relevant WM item. Conversely, an insufficient reduction in beta power might reflect insufficient or ineffective deprioritization of the uncued item and thus predict its decoding remaining high. To test this possibility, we repeated the analysis but with trial-by-trial decoding of the uncued item. Decoding of uncued orientations was highly significant on both Switch (1–1.4 s after cue onset, corrected *p* = 0.0001) and Repeat trials (1.05–1.4 s, *p* = 0.0028, Switch > Repeat 1.25–1.4 s, *p* = 0.0295; Fig. 5*A*, left panel inset). Nevertheless, beta power had no effect on decoding (main effect: *F*_(6.29,264.14)_ = 0.72, *p* = 0.64, Switch trials: *t*_(42)_ = 0.66, *p* = 0.51, Repeat trials: *t*_(42)_ = 0.78, *p* = 0.44; Fig. 5*A*). This remained true after regressing out the effect of item distance (all *p* > 0.51; Fig. 5*A*, right panel). Theta power also did not predict decoding of the uncued item (main effect: *F*_(5.91,248.24)_ = 0.319, *p* = 0.92, Switch trials: *t*_(42) _= −1.06, *p* = 0.30, Repeat trials: *t*_(42) _= −0.56, *p* = 0.58; Fig. 5*B*). Regressing out the effect of item distance did not change this result (all *p* > 0.20).

A related possibility is that insufficient beta desynchronization on Switches leads to lingering activation of the uncued item in the representational format of a cued item (i.e., in a prioritized state). To test this, we trained a decoder on cued WM items and tested it on uncued items. We again found no significant effect. Cross-decoding of uncued orientations (training on cued orientations) was significant on both Switch (0.95–1.4 s after cue onset, corrected *p* = 0.0001) and Repeat trials (0.95–1.4 s, *p* = 0.0002, Switch > Repeat n.s.; Fig. 5*C*, left panel inset). Beta power had no effect on decoding (main effect: *F*_(6.15,258.32)_ = 1.78, *p* = 0.10, Switch trials: *t*_(42)_ = 0.05, *p* = 0.96, Repeat trials: *t*_(42) _= −1.26, *p* = 0.21; Fig. 5*C*). This remained true after regressing out the effect of item distance (all *p* > 0.25; Fig. 5*C*, right panel). Theta power also did not predict decoding of uncued orientations (main effect: *F*_(6.14,258.03)_ = 0.418, *p* = 0.87, Switch trials: *t*_(42) _= −0.95, *p* = 0.35, Repeat trials: *t*_(42)_ = 0.80, *p* = 0.43; Fig. 5*D*). Regressing out the effect of item distance did not change this result (all *p* > 0.41; Fig. 5*D*, right panel). Alpha power again had no effect on decoding of the uncued item (Fig. S2*d*,*e*).

We conclude that beta power reduction affects only the activation of a newly cued item but not the removal of a previously relevant item from the prioritized representational state.

## Discussion

Our study examined the role of neural oscillations in priority switches in working memory. Previous studies had identified predominantly theta and alpha oscillations, with relatively less emphasis on beta-band oscillations, but their respective contributions have remained unclear. Here we identified a central role for transient reductions in beta-band power in two aspects of switching priority in working memory: the magnitude of the representational shift and the fidelity of the update, as measured by the decoding strength of the newly prioritized item. In contrast, while priority switches also affected theta and alpha power, neither of those oscillations strongly correlated with update magnitude or fidelity.

At the behavioral level, priority switches incurred a temporary cost that manifested in reduced accuracy and increased reaction time, consistent with classic work on task switching (Monsell, 2003; Koch et al., 2018). The reduction in accuracy was linked to an increase in guess rate but no change in precision, suggesting that switch costs may have arisen because of occasional failures to prioritize but not because the representation itself was noisier. In line with this interpretation, switches also increased the rate of swap errors. Interestingly, the cost of switching priorities between memories scaled linearly with the angular distance between the two items. This was a surprisingly strong effect that explained the entirety of the switch cost on accuracy (although swap errors may have contributed to part of this effect). The dependence of switch costs on memory similarity suggests that updating the focus of attention is a graded operation, akin to mental rotation (Shepard and Metzler, 1971). This finding appears to be at odds with models of WM as a storage system for arbitrary content (Bouchacourt and Buschman, 2019; Manohar et al., 2019; Bocincova et al., 2022) since it suggests that control operations in WM reflect the representational space of features in WM. A comparable similarity effect has recently been reported for switch costs between different task rules (Bustos et al., 2024). Cognitive control operations more generally may therefore depend on the representational geometry of task-relevant features, consistent with reports of map-like representations for other cognitive functions (Bellmund et al., 2018).

At the neural level, we observed a robust beta-band (∼15–25 Hz) power reduction following switch cues, particularly at central-parietal electrodes. Power reduction scaled with item distance, suggesting that beta oscillations reflect the magnitude of the required switch. Notably, this effect was specific to Switch trials and absent (or modestly reversed) on Repeat trials, while behavior on Repeat trials still showed an effect of item distance. This specificity to switch trials suggests a role for beta in the switching process, rather than coding for item distance per se. It is unclear why switches between more dissimilar items require a greater reduction in beta amplitude. One possibility is that beta tracks the duration of the switching process, and switching to a more dissimilar item takes longer (as with mental rotation). More dissimilar items could also be encoded in more spatially distant populations, possibly requiring stronger beta reduction (Lundqvist et al., 2023) during a switch. We stress, however, that these are speculative explanations that cannot be resolved with the current data.

Importantly, beta power on priority switches also predicted the fidelity of the updated representation. On Switch trials, lower beta power was associated with stronger decoding of the newly prioritized item later in the decision phase. The correlation was specific to beta frequencies, to the cued (but not the uncued) item, and occurred only on Switch trials. This suggests that beta oscillations are involved in the selection and prioritization of relevant memory items rather than the removal of deprioritized items. This conclusion appears to contrast with previous reports of beta oscillations playing a key role in clearing items from WM when they are no longer needed (Lundqvist et al., 2018). In our paradigm, however, items were not fully removed from memory, as uncued items could still be probed later in the block. Beta oscillations may therefore play distinct roles in updating and clearing WM (Lundqvist et al., 2023, 2024). Beta-band desynchronization has previously been implicated in increasing the coding capacity of neural populations (Hanslmayr et al., 2012) to aid reinstatement of long-term memories (Griffiths et al., 2019), consistent with the proposed role in updating in our task. We also observed a temporal progression of events, with beta desynchronization occurring before probe onset but affecting decoding after probe onset. While it is possible that this was simply because stronger decoding after the probe made it easier to find a significant correlation, it may hint at a temporal sequence in which a reduction in beta power initiates a preparatory cascade of events from identifying and accessing the cued item to then prioritizing it before this is reflected in improved decoding when the cued item is needed for decision-making. We speculate that reductions in beta bursts may not only serve to prepare for upcoming external updates of WM (Liljefors et al., 2024) but also for internal updates within the focus of attention. Consistent with this view, a related line of work has suggested that beta synchronization helps in the selection and downstream routing of behaviorally relevant neural ensembles (Salazar et al., 2012; Spitzer and Haegens, 2017; Rassi et al., 2023).

While the limited spatial resolution of EEG prevents us from drawing strong conclusions about anatomical sources of the beta-band effect, its well-documented involvement in regulating activity between cortex and the basal ganglia (Engel and Fries, 2010; Jenkinson and Brown, 2011; Brittain and Brown, 2014; Schmidt et al., 2019) suggests beta synchrony in corticostriatal loops could orchestrate WM prioritization. Along these lines, the basal ganglia have been proposed as a source of selecting from WM for the next action (Hazy et al., 2006; Chatham et al., 2014). Other work has suggested a direct role for beta desynchronization in the motor system in WM tasks which permit preparation of an action before recall (Schneider et al., 2017; Boettcher et al., 2021). This is unlikely in our case because participants could not anticipate the correct response (probe rotated clockwise/counterclockwise) at the time of the peak beta effect (before probe onset). More broadly, surprise at the less frequent Switch trials (∼30% of trials) could have led to inhibition of the motor system (Wessel and Aron, 2013, 2017), though it is unclear why such an effect would scale with update magnitude or predict the strength of decoding. In sum, while several processes likely occur during prioritization, the specificity of the beta-band effects in our study are most consistent with a role in selecting and prioritizing new information.

Theta oscillations, while elevated on Switch trials, did not scale with item distance and showed no consistent relationship with decoding strength. This suggests that theta may reflect a more general, possibly all-or-none, process signaling control demands rather than the implementation of specific updates. This interpretation is consistent with the apparent role of delta/theta in regulating posterior alpha desynchronization when space-based selection of WM contents is possible (de Vries et al., 2020). It also aligns with a longstanding proposal that midline frontal theta signals cognitive control demands more generally (Cavanagh and Frank, 2014) rather than executing control.

Alpha oscillations, like beta, showed a significant reduction on Switch trials in our study. However, as with theta, we did not observe any correlations with update magnitude or decoding. Spatially lateralized alpha oscillations have previously been linked to successful prioritization of cued WM contents (Poch et al., 2014; Wallis et al., 2015; Myers et al., 2015b; Boettcher et al., 2021). This discrepancy may be due to the nonspatial nature of our task, where items were cued via auditory tones rather than by spatial location. Future work could untangle the contributions of different prioritization mechanisms by comparing spatial with nonspatial selection.

Taken together, our results highlight a previously underappreciated role for beta oscillations in the dynamic reorganization of WM contents. Beta power reduction appears to facilitate the transition of items into the prioritized state, with the degree of reduction reflecting both the magnitude of the update and the quality of the resulting representation. We saw no such relationship with the uncued item, indicating that beta in our task was not directly correlated with deprioritizing previously cued information. However, swap errors were more frequent after priority switches, indicating occasional failures to update the focus of attention, and beta could be relevant to this process or in suppressing the uncued item more generally. While recent work has begun to address the neural mechanisms of swap errors in working memory (Alleman et al., 2024), the role of neural oscillations in this process is unknown and appears to be a fruitful avenue for further research. These findings more broadly highlight the potential relationship between neural oscillations and interference-free maintenance of multiple task representations (Fang et al., 2025). Future work could aim to further dissociate the causal role and specific subprocesses of priority switching supported by beta-band oscillations by examining prioritization efficacy when beta oscillations are entrained artificially or when they are affected by neurological conditions such as Parkinson's disease.
